# The Effect of an Open Carpal Tunnel Release on Thumb CMC Biomechanics

**DOI:** 10.1155/2012/151348

**Published:** 2012-11-20

**Authors:** Marc A. Tanner, Bryan P. Conrad, Paul C. Dell, Thomas W. Wright

**Affiliations:** Department of Orthopaedics and Rehabilitation, Orthopaedics and Sports Medicine Institute, University of Florida, Gainesville, FL 32608, USA

## Abstract

*Purpose*. We have observed worsening thumb pain following carpal tunnel release (CTR) in some patients. Our purpose was to determine the effect of open CTR on thumb carpometacarpal (CMC) biomechanics. *Methods*. Five fresh-frozen cadaver arms with intact soft tissues were used. Each specimen was secured to a jig which fixed the forearm at 45° supination, and the wrist at 20° dorsiflexion, with thumb pointing up. The thumb was axially loaded with a force of 130 N. We measured 3D translation and rotation of the trapezium, radius, and first metacarpal, before and after open CTR. Motion between radius and first metacarpal, radius and trapezium, and first metacarpal and trapezium during loading was calculated using rigid body mechanics. Overall stiffness of each specimen was determined. *Results*. Total construct stiffness following CTR was reduced in all specimens but not significantly. No significant changes were found in adduction, pronation, or dorsiflexion of the trapezium with respect to radius after open CTR. Motion between radius and first metacarpal, between radius and trapezium, or between first metacarpal and trapezium after open CTR was not decreased significantly. *Conclusion*. From this data, we cannot determine if releasing the transverse carpal ligament alters kinematics of the CMC joint.

## 1. Introduction

Carpal tunnel syndrome and basal joint arthritis often coexist [1]. A study of 246 patients with basal joint arthritis of the thumb reported that 39% of the study patients also had carpal tunnel syndrome [2]. Anecdotally, we have observed worsening thumb carpometacarpal (CMC) pain in some patients with previously asymptomatic (or minimally symptomatic) first CMC arthritis after undergoing a carpal tunnel release. The thumb CMC joint is the most common site for reconstruction in the upper extremity secondary to osteoarthritis [3]. It is a semiconstrained joint composed of two saddle-shaped articulations with opposing axes perpendicular to each other. Minimal congruence and bony stability allow for a wide range of motion [4]. 

The anterior border of the carpal tunnel is formed by the transverses carpal ligament (TCL) [5]. The transverse carpal ligament proper inserts into the scaphoid tubercle and trapezial ridge radially and the hamulus and pisiform ulnarly [6]. Studies have noted increase in carpal tunnel volume, increase in the carpal arch width, and an overall decrease in carpal stiffness after carpal tunnel release (CTR) [6–12]. Rotational changes of the hamate, pisiform, and the trapezium have also been reported, as well as an increase in the distance between the trapezium and the hook of the hamate [5]. Previous research suggests a direct relationship between widening of the transverse carpal arch and loss of grip strength [13]. Pillar pain is a common complaint after CTR [14], perhaps as a result of the division of the TCL during surgery [6].

Pain originates most commonly over the pisotriquetral joint, possibly secondary to displacement of the pisiform or alteration of forces over the joint [11, 15]. The same type of biomechanical changes could occur with the trapezium and the CMC joint. The purpose of this study is to determine the effect of open CTR on the kinematics of the trapezium and first CMC joint. Our hypothesis was that a CTR will allow rotation of the trapezium altering the biomechanics of the first CMC joint. These changes could result in the clinical observation of increased first CMC joint pain in patients with previous subclinical CMC arthritis.

## 2. Materials and Methods

**Figure 1:**
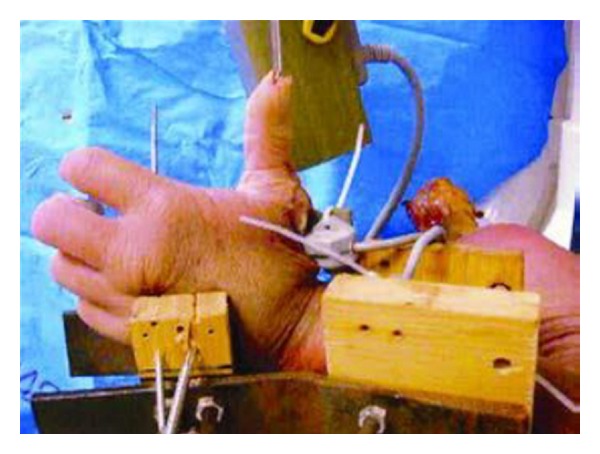
Photograph of a specimen secured in the custom-made jig. The forearm is at 45-degree supination and the wrist at 20-degree dorsiflexion, thumb pointing up.

Five fresh-frozen cadaver arms with intact soft tissues were used. There were three male specimens and two female specimens (donor age was not available). Each specimen was secured to a custom-made jig with 3.0 threaded Steinman pins, two at the distal radius and two placed in the second metacarpal (Figure 1). 

The jig fixed the forearm at 45° supination and was designed to allow free motion at the thumb CMC joint and radiocarpal joint while the wrist was fixed in 20° of dorsiflexion. A threaded 2.0 mm K-wire was placed from the tip of the thumb distal phalanx into the thumb metacarpal, leaving 2 cm of wire out of the skin. The wire out of the skin was placed into an MTS machine (MTS Systems, Eden Prairie, MN) and the jig secured to the base of the testing machine. 

An electromagnetic tracking system (Liberty, Polhemus Inc, Colchester, VT) was used to measure the 3D translation and rotation of the trapezium, radius, and first metacarpal. Electromagnetic sensors were attached to the radius and first metacarpal using custom-made fiberglass brackets. A threaded K-wire was placed into the trapezium, ensuring that the joint capsule remained intact, and a third electromagnetic sensor was attached to the K-wire to track the trapezium. The electromagnetic source was positioned within 20 cm of the sensors to minimize the effect of distortion created by the testing machine and jig. Internal electronics of the Polhemus Liberty system are capable of detecting distortions and, when present, the testing setup was adjusted to eliminate them. After loading at 10 N once to remove any slack, the thumb was axially loaded with a 130 N force. 

The total motion of the first metacarpal relative to the radius, between the radius and trapezium, and between the first metacarpal and the trapezium during loading was calculated using rigid body mechanics. The stiffness of each specimen was also determined by measuring the slope of the load-displacement curve during loading. The measurements were collected from each specimen before and after an open CTR. An open CTR was performed in the standard fashion and verified visually and with palpation. The release was performed without removing the specimen from the jig. A paired *t*-test was used to determine differences in rotation and translation of the specimen befor and after CTR.

## 3. Result

Motion and stiffness data is presented in Table 1. 

All five specimens demonstrated a reduction in the total construct stiffness following CTR; however, the difference was not statistically significant (*P* = 0.1). The average adduction, pronation, and dorsiflexion of the trapezium with respect to the radius did not change significantly after open CRT. No significant decrease in range of motion was measured between the radius and first metacarpal (*P* = 0.12), between the radius and trapezium (*P* = 0.32), or between the first metacarpal and trapezium (*P* = 0.55) after an open CTR.

## 4. Discussion

The purpose of this study was to determine the effect of open CTR on the kinematics of the first CMC joint. Our hypothesis that a CTR would cause rotation of the trapezium altering the biomechanics of the first CMC joint was not supported by the data from this study. Less stiffness was seen in all of the specimens at the radius-metacarpal interface after CTR; however, the difference was not statistically significant. The magnitude of change at the trapezium that would cause symptoms is not known. It may have been that the small changes in stiffness we observed were not statistically significant because the study did not have sufficient power. We do not know what decrease in stiffness would be enough to account for a perceived increase in postoperative pain at the thumb CMC joint in patients with prior subclinical CMC arthritis. Likewise rotation of the trapezium would likely affect the scaphotrapezial trapezoid (STT) joint. This could also cause pain near the base of the thumb. We did not specifically evaluate the STT joint in this study.

The only previous study which addressed this issue was presented in 2009 at the American Academy of Orthopaedic Surgery meeting [16]. Changes in rotation of the trapezium were noted in their study but were not statistically significant. Outward rotational changes in the trapezium of 2.25 degrees (±1.6 degrees) were found after CTR. They also reported rotational changes in the pisiform and the magnitude of these changes was greater than those found at the trapezium (3.83 degrees and 4.5 degrees, resp.). 

A weakness of this study is the small sample size and the inherent variability of cadaveric specimens. Even though the specimens were preconditioned, it is probable that in the normal physiologic state some biomechanical changes may occur over time as the constraints further stretch out. The native musculature was not used to dynamically load the CMC. In a cadaver specimen it is challenging to reproduce physiological loading and it is possible that the tested condition does not reproduce normal CMC loading. The strengths of the study are that the surrounding soft tissues of the forearm were not disturbed and the carpal tunnel release was performed without removing the arm from the custom jig. Many other factors come into play concerning the clinical onset of 1st CMC joint pain including deconditioning or increased activity level once the patient has recovered from carpal tunnel surgery.

Our goal was to explore changes in kinematics at the trapezium following CTR. These changes could be a result of the changes in the anatomic relationship due to the release of the TCL affecting the forces at its insertion onto the trapezial ridge. Small rotational changes could affect the normal kinematics of the CMC joint during physiologic loading. Based on the methodology of this cadaver study, we were unable to prove our hypothesis that releasing the TCL would result in kinematic changes of the trapezium with secondary effects on the 1st CMC joint that could be responsible for postoperative pain in a previous arthritic but asymptomatic CMC joint.

## Figures and Tables

**Table 1:** Summary of mechanical properties for each combination of bones before and after open carpal tunnel release.

Measurement	Intact (mean ± SD)	After release (mean ± SD)	Difference	*P* value
Rad-Met1 *R*_*x*_ (adduction) (deg)	2.2 ± 1.3	2.1 ± 1.6	6.6%	0.69
Rad-Met1 *R*_*y*_ (pronation) (deg)	0.7 ± 0.6	0.7 ± 0.4	−7.9%	0.56
Rad-Met1 *R*_*z*_ (dorsiflexion) (deg)	3.5 ± 1.4	3.4 ± 1.5	3.0%	0.78
Rad-Met1 *X* (dorsal deviation) (mm)	1.9 ± 1.6	2 ± 1.7	−3.3%	0.48
Rad-Met1 *Y* (subluxation) (mm)	1.4 ± 0.7	1.5 ± 0.7	−9.1%	0.12
Rad-Met1 *Z* (ulnar deviation) (mm)	1.5 ± 0.7	1.5 ± 0.6	−5.5%	0.54
Rad-Trap *R*_*x*_ (adduction) (deg)	1.9 ± 1.1	2.1 ± 1.2	−10.2%	0.17
Rad-Trap *R*_*y*_ (pronation) (deg)	2.9 ± 2.2	2.9 ± 2.4	−0.9%	0.83
Rad-Trap *R*_*z*_ (dorsiflexion) (deg)	2.0 ± 2.7	1.9 ± 2.3	4.1%	0.70
Rad-Trap *X* (dorsal deviation) (mm)	1.3 ± 0.9	1.1 ± 0.8	11.0%	0.13
Rad-Trap *Y* (subluxation) (mm)	1.0 ± 1.1	1.0 ± 1.1	−6.0%	0.32
Rad-Trap *Z* (ulnar deviation) (mm)	1.2 ± 0.5	1.3 ± 0.6	−8.6%	0.31
Trap-Met1 *R*_*x*_ (adduction) (deg)	9.0 ± 9.8	8.6 ± 8.8	5.1%	0.60
Trap-Met1 *R*_*y*_ (pronation) (deg)	2.7 ± 1.7	2.6 ± 2.0	2.5%	0.83
Trap-Met1 *R*_*z*_ (dorsiflexion) (deg)	12 ± 11.2	11.9 ± 11.2	1.3%	0.80
Trap-Met1 *X* (dorsal deviation) (mm)	0.5 ± 0.2	0.6 ± 0.5	−32.4%	0.36
Trap-Met1 *Y* (subluxation) (mm)	1.2 ± 1.2	1.2 ± 1.2	−2.8%	0.55
Trap-Met1 *Z* (ulnar deviation) (mm)	0.7 ± 0.4	0.6 ± 0.4	5.3%	0.39
Overall specimen stiffness (N/mm)	48.9 ± 23.4	41.4 ± 18.4	15.3%	0.11

Rad-Met: radius to 1st metacarpal; Rad-Trap: radius to trapezium; Trap-Met: trapezium to 1st metacarpal.
